# Obstetrics

**Published:** 1878-12

**Authors:** 


### Obstetrics.

A New Device for Arresting Post Partum Hæmorrhage. Christie. (*Med. Press & Circular*, Sept. 4, '78.)

This consists of an india-rubber bag of about a pint capacity, having a tube and stop-cock attached. The air is pressed out of the bag, the valve closed, and the former introduced into the uterine cavity. The end of the tube is then opened in a basin of tepid water, at a height of two or three feet above the uterus. By atmospheric pressure it will be filled in a few seconds, and by regulating the height of the water supply, the pressure in the bag can be made to counteract the intra-arterial pressure. By keeping the valve open, the uterus expels from the bag, as it contracts, the requisite amount of water, and if it relax in the after pains, the water readily re-enters, and the pressure is always the same. In post partum cases the outside of the bag should always be smeared with glycerine. The author uses the same instrument in cases of placenta prævia, only filling it with cold instead of tepid water.

